# Relationship between mastication and malnutrition in community-dwelling older adults: a meta-analysis

**DOI:** 10.1590/2317-1782/20242023209en

**Published:** 2024-05-31

**Authors:** Alba Maria Melo Medeiros, Ramon Cipriano Pacheco de Araújo, Allya Francisca Marques Borges, Sara Estéfani Soares de Sousa, Cristiano Miranda de Araujo, Hipólito Virgilio Magalhães, Renata Veiga Andersen Cavalcanti, Karinna Veríssimo Meira Taveira

**Affiliations:** 1 Grupo de Pesquisa Estudos em Motricidade Orofacial e Disfagia Orofaríngea, Universidade Federal do Rio Grande do Norte – UFRN - Natal (RN), Brasil.; 2 Programa de Pós-graduação em Fonoaudiologia, Universidade Federal do Rio Grande do Norte – UFRN - Natal (RN), Brasil.; 3 Programa de Pós-graduação em Ciências da Saúde, Universidade Federal do Rio Grande do Norte – UFRN - Natal (RN), Brasil.; 4 Programa de Pós-graduação em Distúrbios da Comunicação, Núcleo de estudos Avançados em Revisão Sistemática e Meta-análise – NARSM, Universidade Tuiuti do Paraná – UTP - Curitiba, PR, Brasil.; 5 Departamento de Fonoaudiologia, Universidade Federal do Rio Grande do Norte – UFRN - Natal (RN), Brasil.; 6 Programa Associado de Pós-graduação em Fonoaudiologia, Núcleo de Estudos Avançados em Revisão Sistemática e Meta-análise – NARSM, Departamento de Morfologia, Centro de Biociências, Universidade Federal do Rio Grande do Norte – UFRN - Natal (RN), Brasil.

**Keywords:** Older People, Chewing, Masticatory Force, Malnutrition, Systematic Review

## Abstract

**Purpose:**

To determine the relationship between mastication and malnutrition in community-dwelling older adults.

**Research strategies:**

To establish the eligibility criteria, the acronym PECOS was used: Population: non-institutionalized older adults; Exposure: older adults with malnutrition; Control: older adults without malnutrition; Outcome: masticatory problems in malnourished older adults; Study types: observational studies.

**Selection criteria:**

It selected studies assessing malnutrition and mastication difficulties in non-institutionalized adults over 60 years old, of both sexes. Mastication and malnutrition were evaluated with questionnaires on self-reported symptoms and clinical and instrumental assessments. There were no restrictions on language, year of publication, or ethnicity.

**Data analysis:**

The included studies were evaluated for methodological quality with the Joanna Briggs Institute tool for cross-sectional studies. For the cross-sectional studies included, the odds ratio (OR) was calculated with 95% confidence intervals.

**Results:**

After searching the databases, 692 references were retrieved, with three studies selected for quantitative and qualitative analysis. The values obtained in the meta-analysis for association show that malnutrition and mastication difficulties were 2.21 times as likely to occur (OR = 2.21; 95%CI = 0.93 – 5.26; I2 = 94%) as individuals without malnutrition (p < 0.001). The assessment of the risk of bias presented a high-risk, a moderate-risk, and a low-risk study. The certainty of evidence was rated very low with the GRADE tool.

**Conclusion:**

Individuals at risk of malnutrition are 2.21 times as likely to have mastication difficulties.

## INTRODUCTION

People over 65 years old are among the fastest-growing age groups. By 2050, one in every six people worldwide is expected to be over 65 years old (16%), up from one in eleven in 2019 (9%)^(1)^. The increase in life expectancy highlights the need to pay closer attention to this population’s health.

Nutritional status is an important health factor in older patients, and it must be assessed to prevent numerous acute and chronic diseases^(2)^. Oral disorders associated with reduced mastication negatively affect older adults’ nutritional status^(3)^. The inability to properly chew and grind food tends to exclude some staple foods from their diets, such as meats, fruits, and vegetables, favoring the consumption of refined carbohydrates, fats, or soft or overcooked foods that pose a risk of missing micronutrient sources^(4)^. Malnutrition can be defined as a nutritional status resulting from the absence of ingestion or disturbance in the absorption of nutrients. It is characterized by deficits in lean and fat mass in the body composition in relation to the person's height. Hence, it decreases physical and mental functions, aggravating their clinical condition^(5)^.

The oral cavity, in the masticatory process, is the gateway to the consumption of nutrients^(6)^. As stated by Yoshida et al.^(7)^, tooth loss leads to a change in diet and may, therefore, be linked to eating disorders, such as obesity and malnutrition. Given the evidence in the literature^(8)^, it is clear that reduced masticatory performance is associated with nutritional disorders. This may be linked to the fact that reduced masticatory function leads the subject to consume a greater amount of soft foods, including foods rich in fats or refined carbohydrates, and to reduce the intake of fruits, vegetables, and meat. Thus, the present study aimed to carry out a systematic review relating mastication with malnutrition in community-dwelling older adults.

## METHODS

### Protocol

This systematic review was performed according to the PRISMA^(9)^ (Preferred Reporting Items for Systematic Reviews and Meta-Analyses) guidelines and used the methods recommended by the Cochrane Manual for Systematic Reviews.

### Register

The protocol of this systematic review was registered on PROSPERO – International prospective register of systematic review – Center for Reviews and Dissemination University of York – CRD42022315585^10^.

### Search strategy and study selection

To establish the eligibility criteria, the acronym PECOS was used: Population: non-institutionalized older adults; Exposure: older adults with malnutrition; Control: older adults without malnutrition; Outcome: masticatory problems in malnourished older adults; Study types: observational studies.

The present study sought to observe the relationship between masticatory problems and malnutrition in community-dwelling older adults. Thus, it selected studies assessing malnutrition and mastication difficulties in non-institutionalized adults over 60 years old, of both sexes. Mastication and malnutrition were evaluated with questionnaires on self-reported symptoms and clinical and instrumental assessments. There were no restrictions on language, year of publication, or ethnicity.

The following exclusion criteria were applied: 1. Studies that did not assess mastication; 2. Studies that did not assess malnutrition; 3. Studies in institutionalized or hospitalized older adults, or conducted in community health centers or primary health care; 4. Studies in children, adolescents, and adults up to 60 years old; 5. Studies in which it was not possible to establish a relationship between mastication and malnutrition; 6. Reviews, letters, books, congress abstracts, case reports, case series, opinion articles, technical articles, and guidelines; 7. Studies unavailable for full-text reading.

Word combinations were adapted for each of the six databases selected as sources of information: EMBASE, PubMed/MEDLINE, Latin American and Caribbean Health Sciences Literature (LILACS), Scopus, and Web of Science. The grey literature was also used as a source of information through Google Scholar, OpenGrey, and ProQuest. The databases and grey literature were searched on January 19, 2022, and updated on August 3, 2023; references were managed, and duplicates were removed using appropriate Mendeley software (Elsevier Inc., New York, NY). The search strategy used can be found in Chart 1.

**Chart 1:** Database search strategy

**Database**	**Search (January 19^th^ 2022, and update August 3^th^ 2023)**
**Embase**	'aged'/exp OR 'aged' OR 'elderly'/exp OR 'elderly' OR 'elderlies' OR 'elderly people'/exp OR 'elderly people' OR 'elderly population' OR 'aging'/exp OR 'aging' OR 'ageing'/exp OR 'ageing' OR 'older adults'/exp OR 'older adults' OR 'senior' OR 'seniors' OR 'geriatric'/exp OR 'geriatric' OR 'geriatrics'/exp OR 'geriatrics' AND 'malnutrition' OR 'nutritional deficiency' OR 'nutritional deficiencies' OR 'undernutrition' OR 'malnourishment' OR 'weight loss' OR 'management of malnutrition' AND 'mastication' OR 'chewing' OR 'chewing ability' OR 'chewing performance' OR 'masticatory ability' OR 'masticatory function' OR 'masticatory disability' OR 'masticatory dysfunction' OR 'chewing function' OR 'chewing discomfort' OR 'chewing disorder' OR 'chewing problems'
**LILACS**	“Idosos” OR “Pessoa de Idade” OR “Pessoa Idosa” OR “Pessoas de Idade” OR “Pessoas Idosas” OR “População Idosa” “Anciano” OR “Adulto Mayor” OR “Ancianos” OR “Persona de Edad” OR “Persona Mayor” OR “Personas de Edad” OR “Personas Mayores” OR “Aged” OR “elderly” OR “elderlies” OR “elderly's” OR “elderly people” OR “elderly population” OR “Aging” OR “ageing” OR “older adults” OR “senior” OR “seniors” OR “geriatric” OR “geriatrics” AND “Desnutrição” OR “Desnutrición” OR “Malnutrition” OR “nutritional deficiency” OR “nutritional deficiencies” OR “undernutrition” OR “malnourishment” OR “weight loss” OR “management of malnutrition” OR “Subalimentação” OR “Subnutrição” AND “Mastigação” OR “Masticación” OR “Mastication” OR “chewing” OR “chewing ability” OR “chewing performance” OR “masticatory ability” OR “masticatory function”
**PubMed/ Medline**	“Aged” OR “elderly” OR “elderlies” OR “elderly's” OR “elderly people” OR “elderly population” OR “Aging” OR “ageing” OR “older adults” OR “senior” OR “seniors” OR “geriatric” OR “geriatrics” AND “Malnutrition”[MeSH Terms] OR “Malnutrition” OR “nutritional deficiency” OR “nutritional deficiencies” OR “undernutrition” OR “malnourishment” OR “weight loss” OR “management of malnutrition” AND “Mastication”[MeSH Terms] OR “Mastication” OR “chewing” OR “chewing ability” OR “chewing performance” OR “masticatory ability” OR “masticatory function” OR “masticatory disability” OR “masticatory dysfunction” OR “chewing function” OR “chewing discomfort” OR “chewing disorder” OR “chewing problems”
**Scopus**	(TITLE-ABS-KEY (“Aged” OR “elderly” OR “elderlies” OR “elderly's” OR “elderly people” OR “elderly population” OR “Aging” OR “ageing” OR “older adults” OR “senior” OR “seniors” OR “geriatric” OR “geriatrics”) AND TITLE-ABS-KEY (“Malnutrition” OR “nutritional deficiency” OR “nutritional deficiencies” OR “undernutrition” OR “malnourishment” OR “weight loss” OR “management of malnutrition”) AND TITLE-ABS-KEY (“Mastication” OR “chewing” OR “chewing ability” OR “chewing performance” OR “masticatory ability” OR “masticatory function” OR “masticatory disability” OR “masticatory dysfunction” OR “chewing function” OR “chewing discomfort” OR “chewing disorder” OR “chewing problems”))
**Web of Science**	“Aged” OR “elderly” OR “elderlies” OR “elderly's” OR “elderly people” OR “elderly population” OR “Aging” OR “ageing” OR “older adults” OR “senior” OR “seniors” OR “geriatric” OR “geriatrics” (Tópico) and “Malnutrition” OR “nutritional deficiency” OR “nutritional deficiencies” OR “undernutrition” OR “malnourishment” OR “weight loss” OR “management of malnutrition” (Tópico) and “Mastication” OR “chewing” OR “chewing ability” OR “chewing performance” OR “masticatory ability” OR “masticatory function” OR “masticatory disability” OR “masticatory dysfunction” OR “chewing function” OR “chewing discomfort” OR “chewing disorder” OR “chewing problems” (Tópico)
**Google Scholar**	“older adults” AND “Malnutrition” AND “chewing problems” OR “masticatory disability” filetype:PDF
**Biblioteca digital brasileira de teses e dissertação (BDTD)**	“Idosos” OR “Pessoa de Idade” OR “Pessoa Idosa” OR “Pessoas de Idade” OR “Pessoas Idosas” OR “Aged” OR “elderly” OR “elderlies” OR “elderly's” OR “elderly people” OR “elderly population” OR “Aging” OR “ageing” OR “older adults” OR “senior” OR “seniors” OR “geriatric” OR “geriatrics” AND “Desnutrição” OR “Malnutrition” OR “nutritional deficiency” OR “nutritional deficiencies” OR “undernutrition” OR “malnourishment” OR “weight loss” OR “management of malnutrition” OR “Subalimentação” OR “Subnutrição” AND “Mastigação” OR “Mastication” OR “chewing” OR “chewing ability” OR “chewing performance” OR “masticatory ability” OR “masticatory function”
**Open Gray**	“older adults” AND “Malnutrition”
**ProQuest**	noft((“aged” OR “elderly” OR “elderly people” OR “elderly population” OR “elderlies” OR “elderly's” OR “elderlys” OR “aging” OR “ageing” OR “senescence” OR “older adults” OR “older adult” OR “older people” OR “senior” OR “seniors” OR “geriatric” OR “geriatrics”)) AND noft((“myofunctional therapy” OR “myofunctional therapies” OR “orofacial myotherapy” OR “oral myotherapy” OR “orofacial myology” OR “oral exercise” OR “promotion program” OR “long-term care prevention programs” OR “resistance training” OR “strength training” OR “exercise program” OR “exercise therapy” OR “remedial exercise”)) AND noft((“Stomatognathic System” OR “Masticatory System” OR “mastication” OR “Chewing” OR “Deglutition” OR “Deglutitions” OR “Swallowing” OR “Swallowings” OR “Speech” OR “Respiration” OR “Breathing”))

### Data extraction

Articles were selected in two stages. In the first one, two reviewers (AMMM and RCPA) independently reviewed the titles and abstracts of all references. All articles that did not meet the previously established eligibility criteria were excluded at this stage. In the second one, the same reviewers read the full text of the articles selected in the first stage, also independently. When they disagreed, and there was no consensus even after discussion, a third reviewer (RVAC) was included in the final decision. To ensure greater agreement between the reviewers, they were calibrated with part of the selected literature using a sample of 100 titles/abstracts before beginning stage 1. Hence, full-text reading only started after a value > 0.7 was obtained in the kappa agreement coefficient.

Two reviewers (AMMM and RCPA) independently collected information from the included articles. The following data were collected: study characteristics (author, year of publication, country, and study design), sample characteristics (sample type and size), nutritional assessment, mastication assessment, outcomes of the relationship between mastication and nutrition, and whether the sample lacked teeth. When data were missing or incomplete in the article, attempts were made to contact the authors to obtain pertinent unpublished information.

### Assessment of methodological quality

The included studies were evaluated for methodological quality with the Joanna Briggs Institute^(14)^ tool for cross-sectional studies. Two reviewers (AMMM and RCPA) separately assessed the risk of bias and judged the included articles, marking each evaluation criterion with “yes”, “no”, “uncertain”, or “not applicable”. The risk of bias was classified as high when the study reached 49% “Yes”; moderate when the study reached 50% to 69% “Yes”; and low when the study reached more than 70% “Yes”^(15)^. When necessary, disagreements were solved by discussing with a third reviewer (KVMT). Revman 5.4 software (Review Manager 5.4; The Cochrane Collaboration) was used to generate the figures.

Results were analyzed with GRADE® (Classification of Recommendations, Assessment, Development and Evaluation)^(16)^, which is a quality scoring system for results. Two reviewers (AMMM and RCPA) judged the following aspects: risk of bias, inconsistency, imprecision, indirect evidence, and publication bias. Disagreements were solved by consensus with the third reviewer (KVMT). The level of evidence was classified as high, moderate, low, or very low.

### Statistical analysis

The number of events and the sample size were collected from each study to calculate the measure of association. For the cross-sectional studies included, the odds ratio (OR) was calculated with 95% confidence intervals (CI).

The odds ratio estimates were calculated with the Mantel-Haenszel method to assess the association between malnutrition and mastication difficulties^(17)^. Considering the observational study design and the possible methodological or clinical heterogeneity causing a degree of variance in the measures of interest, the combined estimates were obtained with the random effects models. The heterogeneity between the studies grouped in the analysis was evaluated with the Higgins I^2^ statistics^(18)^. The forest plot was generated with the Review Manager 5.4® software (RevMan 5.4, Copenhagen, Denmark), and the significance level was set at 5%.

If possible (n > 10), publication bias would be assessed with funnel plots. However, as this assessment was unfeasible, a broad search strategy was carried out in databases and the grey literature, besides consulting an expert for unpublished articles, in order to reduce the risk of publication bias.

## RESULTS

### Selection of studies

**Figure 1:**
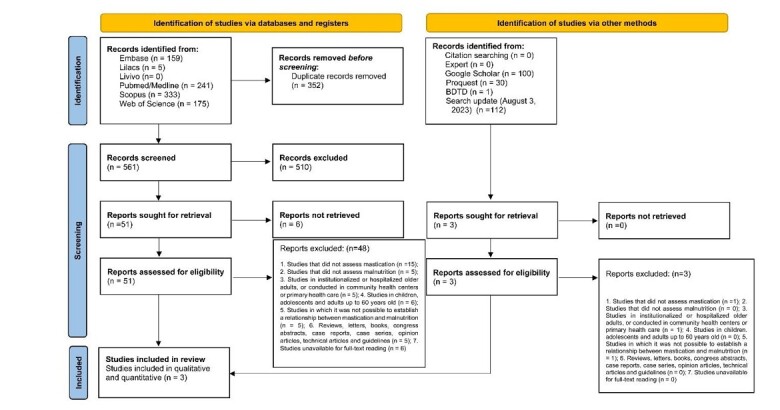
Flow diagram of the literature search and selection criteria^9^

The search strategy found 1,044 studies in six databases and the grey literature. After removing the duplicates, 804 studies remained for analyzed. Based on the title and abstract reading (stage 1), 750 articles were removed in the first search. The remaining ones were assessed for eligibility by full-text reading (stage 2), in which 51 were removed (Appendix A). No studies were added to the reference list after consultation with an expert and updating the search. Hence, three studies were included in the quantitative and qualitative analysis (Figure 1).

### Characteristics of the studies

Regarding age, one study considered older adults those 60 years or older^(11)^, following the World Health Organization (WHO) guidelines for developing countries. Two other studies considered those 65 years or older^(12,13)^. The samples consisted of 294 (57.8%) women and 215 (42.2%) men in one survey,^(12)^ 1,591 (50.7%) men and 1,543 (49.2%) women in another^(13)^, and 363 (36.1%) men and 640 (63.8%) women in the other study^(11)^.

All three studies are cross-sectional. Regarding the country, two studies were carried out in Japan^(12,13)^ and the other one was carried out in India. The samples ranged from 980^(11)^ to 3,141^(13)^. The study characteristics are described in Table 1.

**Table 1:** Summary of the characteristics of the studies included (n=3)

**Author, year, and country**	**Sample size and sex**	**Mean ages**	**Nutritional assessment**	**Mastication assessment**	**Result of the relationship between mastication and nutrition**	**Lacking/not lacking teeth**
Gupta et al.^(11)^, 2019, India	n=1,003*; 363 men; 640 women;	69.5 years (men); 67.8 years (women)	Mini Nutritional Assessment, 24-hour dietary recall (n = 248), and body mass index (BMI)	Oral examination	Malnourished with mastication problems: 26.4% (88)	Malnourished, completely toothless = 13 (24.5). Malnourished, with teeth = 127 (13.7)
					No mastication problem: 8% (52)	
						At risk of malnutrition, completely toothless = 34 (64.2)
						At risk of malnutrition, with teeth = 587 (63.3)
					Malnutrition prevalence (MNA < 17) was 14.3%	
Motokawa et al.^(12)^, 2021, Japan	n= 509 older adults; 294 women	301 participants had poor mastication (73.9 years); 208 had good mastication (72.1 years)	Blood sample; Anthropometric measures; semi-quantitative food frequency questionnaire (Takahashi et al., 2001)	Chewing gum test (Xylitol; Lotte, Saitama, Japan)	301 participants (59.1%) had poor mastication, and 208 had good mastication (40.9%)	Older adults with mastication problems and mean number of teeth: 26.3 (±4.1) p= <0.001
						Older adults without mastication problems and mean number of teeth: 27.6 (±1.8) p= <0.001
					60 participants (11.8%) were malnourished, while 449 (88.2%) had a good nutritional status	
					Masticatory ability and nutritional status (Model 1): 95%CI: 1.205- 1.994 p= <0.001	
					Masticatory ability and nutritional status (Model 2): 95%CI: 1.004–2.022 p= 0.027	
Okamoto et al.^(13)^, 2019, Japan	n = 1,591 men; 1,543 women	71 years	Blood sample and BMI	Self-report questionnaire and maximum occlusal force measure (Dental Prescale System)	Able to masticate all food groups; with serum albumin <4.4: 260 (20.5%) p=0.041	All five groups of chewable foods significantly decreased with the number of teeth (p= <0.001)
					Unable to masticate all food groups; with serum albumin <4.4: 83 (25.8%) p= 0.041	

Caption: BMI = body mass index; MNA = Mini Nutritional Assessment; *n = 980 actually participated

### Risk of study bias

**Figure 2:**
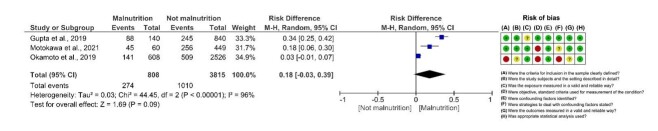
Forest plot of the meta‐analysis of the relationship between mastication and malnutrition in community-dwelling older adults, and displaying risk-of-bias judgements for each study included

One study^(13)^ was assessed with a high risk of bias, one study^(11)^ with a moderate risk, and the other one^(12)^ with a low risk. The results of the risk of bias are described in Figure 2 and Chart 2.

**Chart 2:** Risk of bias assessed by the Joanna Briggs Institute critical appraisal tool for cross-sectional studies

Question	Answer*		
	Gupta et al.^(11)^	Motokawa et al.^(12)^	Okamoto et al.^(13)^
1. Were the inclusion criteria in the sample clearly defined?	Y	Y	N
2. Were the study subjects and the scenario described in detail?	Y	Y	U
3. Was the exposure measured in a valid and reliable way?	U	Y	Y
4. Were objective and standard criteria used to measure the condition?	N	N	N
5. Were confounding factors identified?	N	Y	Y
6. Were strategies for deal with confounding factors stated?	N	U	N
7. Were the outcomes measured in a valid and reliable way?	Y	Y	U
8.Was appropriate statistical analysis used?	Y	Y	Y
% yes/risk	50%	75%	37.5%
	Mod	Low	Hight

* Y = Yes; N = No; U = Unclear

### Results of individual studies

Gupta et al.^(11)^ evaluated malnutrition and mastication in older adults living in 30 communities by assessing their oral status, body mass index, and 24-hour dietary recall and applying the Mini Nutritional Assessment with previously trained researchers. Masticatory capacity was analyzed through oral examinations and interviews. During the oral examination, subjects were requested to open their oral cavities for inspection. A comprehensive examination of the teeth ensued, involving the counting of lost teeth. Edentulous status was established when no teeth were present in both the upper and lower jaws. Palpation of the lower face was carried out to assess missing teeth and edentulous status, while subjects were also examined for the presence of any chronic oral diseases that could impact dental health. They found that 26.4% were malnourished with mastication problems, 8.0% were malnourished without mastication problems.

Motokawa et al.^(12)^ examined the cognitive functions of older adults in urban communities to enhance their engagement in the research aimed at assessing serum albumin levels. In addition, anthropometric measurements and semi-quantitative data on food frequency were collected. Malnutrition was categorized based on the cutoff value of 4.0 g/dL in albumin analysis. Mastication capacity was evaluated using xylitol color-changing chewing gum; if it turned green within 1 minute, it indicated a diminished mastication capacity. The researchers identified that 8.8% of the participants demonstrated both malnutrition and mastication problems, while 2.9% were malnourished but did not experience mastication difficulties.

Okamoto et al.^(13)^ verified the relationship between tooth loss, low masticatory capacity, and nutritional indices in community-dwelling older adults. Objective masticatory evaluation was employed to measure occlusal force. In terms of nutritional assessment, body mass index was recorded, and blood samples were collected following an overnight fast to determine serum albumin levels. The masticatory capacity was assessed using the Dental Prescale System^(19,20)^, which employs microcapsules embedded in a horseshoe-shaped pressure-sensitive sheet with layered colors. These microcapsules rupture based on occlusal pressure, leading to a red coloration due to chemical reactions. The authors found that 4.4% were malnourished with mastication problems and 14.9% were malnourished without mastication problems.

### Summary of results

The results of the meta-analysis regarding the association indicate that the likelihood of experiencing malnutrition and mastication difficulties was 2.21 times higher (OR = 2.21; 95%CI = 0.93 – 5.26; I^2^ = 94%) compared to individuals without malnutrition (Figure 2).

### Reporting bias

As it was not possible to assess with a funnel plot (n˂10) to reduce the probability of publication bias, a wide search was carried out in several databases, including a non-English language base (LILACS), and in the grey literature.

### Level of evidence

The certainty of evidence was rated very low with the GRADE® system. This result was due to the assessment of risks of bias of the included studies, unclear exclusion criteria, uncontrolled confounding factors, wide confidence interval, and high heterogeneity (Table 2).

**Table 2:** Analysis of information quality through GRADE

**Research question:** To determine the relationship between mastication and malnutrition in community-dwelling older adults?							
**Certainty assessment**							**Certainty**
**Nº of studies**	**Study design**	**Risk of bias**	**Inconsistency**	**Indirectness**	**Imprecision**	**Other considerations**	**Prevalencia**
3	observational studies	Serious^a^	serious^b^	not serious	Serious^c^	none	⨁◯◯◯
							VERY LOW

*Explanations*

a Exclusion factors were not undefined, and confounding factors;

b Presence of a source of heterogeneity even after the feasibility analysis;

c Wide confidence interval

## DISCUSSION

This systematic review aimed to gather available evidence regarding the relationship between malnutrition and mastication in community-dwelling older. The outcome of the meta-analysis conducted in this study revealed that individuals facing mastication difficulties exhibited a 2.21-fold higher likelihood of experiencing malnutrition. The certainty of evidence was classified as very low with the GRADE® system, which is explained by the assessment of the risk of bias of the studies (classified as high^(13)^, moderate^(11)^, and low risk^(12)^), uncontrolled confounding factors, wide confidence intervals, and high heterogeneity. The sample size varied widely, from 509 participants in one study^(11)^ to 3,134 participants in a survey^(13)^. Due to the differences in the samples, measure assessments, and outcome tools, the meta-analysis had 94% of heterogeneity. Hence, it was not possible to identify the reason with a sensitivity test because of the few studies and great methodological and clinical variability.

All the studies included in this analysis were cross-sectional and focused on investigating the impact of masticatory problems on the subjects’ nutritional status. It is important to recognize that the results presented by cross-sectional studies do not allow for the determination of cause-and-effect relationships^(19)^. However, these cross-sectional analyses are commonly performed to determine factors that are associated with a particular health problem – in this case, malnutrition. All studies had some degree of methodological flaws and some kind of bias. A common issue observed among them was the omission of confounding factors an example is socioeconomic status. The socioeconomic background of individuals can markedly influence both the prevalence of chewing problems and nutritional status. Individuals with lower socioeconomic status may face obstacles in accessing proper dental care or nutrition, introducing potential bias into the observed association. Overlooking these confounding factors hampers a comprehensive understanding of the intricate interplay between chewing problems and nutritional outcomes, which can potentially distort the accurate assessment of how an exposure influences an outcome.

This is the first systematic review of the relationship between mastication and malnutrition in non-institutionalized older adults over 60 years of age. The strengths of this review include unrestricted literature searches, no language limitations, and studies independently selected by two researchers. However, there is still no consensus in the literature on mastication assessment, which can be performed in different ways. In this study, the masticatory assessment was performed with physical examinations by trained professionals^(11)^, using color-changing chewing gum^(12)^, maximum occlusal force measurement, and a self-reported questionnaire^(13)^.

It is worth noting that even self-reported questionnaires are tools widely used in research – even though they may pose a memory bias, especially if they are not validated, impacting the result of the risk of bias. However, only one article included used a nutritional self-reference questionnaire, which is validated and widely used in research and clinical practice. Furthermore, a significant strength observed in the included studies was the consensus in nutritional assessments. These assessments were conducted meticulously, with careful avoidance of evaluations conducted shortly after a period of hospitalization. This precaution was taken to minimize the potential for bias.

To better understand the relationship between mastication and malnutrition, it is important to point out that oral disorders associated with reduced mastication negatively affect the nutritional status of older adults^(3)^. In this review, a study^(11)^ shows that completely toothless individuals with poor oral health status and mastication problems had significantly poor nutritional status and body mass index (BMI). Another study^(13)^ presents the same finding, thus corroborating the findings in the literature^(20)^.

Oral health is a seemingly neglected public health problem, in spite of its relevance. It is estimated that 3.5 billion people worldwide suffer the consequences of untreated oral conditions^(21)^. Many changes in masticatory function are subtle but can enhance age-related pathological processes. As healthy diets can have systemic beneficial effects, oral care also plays an important role in maintaining and improving not only oral health but also general health and well-being^(22)^.

Motokawa et al.^(12)^ also demonstrate that in the group with low chewing capacity, all levels of nutrient intake were significantly low, except for carbohydrates, which confirms the findings in the literature^(4)^. Therefore, it is demonstrated that the inability to properly chew and grind food tends to exclude some staple foods from their diets, giving priority to the consumption of refined carbohydrates. Dental status can also affect the choice and preparation of food; when they are no longer able to cut and chew food properly, they end up opting for easy-to-eat foods, which are often less nutritious. Thus, they tend to exclude foods that require a greater masticatory demand. According to the included studies^(11-13)^, a low number of teeth is significantly correlated with low masticatory capacity.

Given the demographic changes that began in the 20th century, with increasingly older populations, it is important to ensure older adults not only a longer survival but also a good quality of life^(1)^. There were limitations in this systematic review, such as the different methodologies used, wide confidence intervals, different diagnostic criteria and sample size, and the few studies available with associations between mastication and malnutrition. Hence, further studies should be carried out to examine the effects of mastication on malnutrition with better methodological quality, avoiding the limitations found in this review.

## CONCLUSION

The values obtained in the meta-analysis showed that individuals at risk of malnutrition are 2.21 times as likely to have mastication difficulties. The results indicate that malnutrition is associated with mastication difficulties. However, given the level of certainty, the results should be cautiously evaluated, and studies with better methodological quality are suggested.

## Appendix A Excluded articles and reasons for exclusion (n = 51)

**Table:**

**Author**	**Reason for exclusion** *
Alia et al.^(1)^	3
Aquilanti et al.^(2)^	2
Baumgartner et al.^(3)^	4
Braud et al.^(4)^	4
Borges et al.^(5)^	4
Brodeur et al.^(6)^	2
Chatindiara et al.^(7)^	1
Cousson et al.^(8)^	1
Harel et al.^(9)^	3
Hutton et al.^(10)^	5
Iwasaki et al.^(11)^	5
Khandelwal et al.^(12)^	6
Khandelwal et al.^(13)^	1
Kiesswette et al.^(14)^	6
Kim et al.^(15)^	1
Kim et al.^(16)^	1
Kiss et al.^(17)^	1
Kolb et al.^(18)^	6
Krishna et al.^(19)^	1
Kwon et al.^(20)^	2
Majumder et al.^(21)^	1
Mann et al.^(22)^	2
Manzon et al.^(23)^	1
Matsuo et al.^(24)^	7
Mbodj et al.^(25)^	4
Morais et al.^(26)^	1
Musacchio et al.^(27)^	5
Oliveira et al.^(28)^	7
Oliveira et al.^(29)^	1
Osta et al.^(30)^	3
Osta et al.^(31)^	2
Özsürekci et al.^(32)^	7
Özsürekci et al.^(33)^	3
Palmer et al.^(34)^	1
Putri et al.^(35)^	3
Riesen et al.^(36)^	6
Saksono et al.^(37)^	1
Saletti et al.^(38)^	1
Samnieng et al.^(39)^	5
Samnieng et al.^(40)^	5
Semba et al.^(41)^	5
Soini et al.^(42)^	1
Stajkovic et al.^(43)^	6
Tani et al.^(44)^	5
Treesattayakul et al.^(45)^	4
Volkert et al.^(46)^	7
Volkert et al.^(47)^	7
Woo et al.^(48)^	1
Yuniendra et al.^(49)^	3
Wallace et al.^(50)^	4
Ziylan et al.^(51)^	1

* 1. Studies that did not assess mastication; 2. Studies that did not assess malnutrition; 3. Studies in institutionalized or hospitalized older adults, or conducted in community health centers or primary health care; 4. Studies in children, adolescents, and adults up to 60 years old; 5. Studies in which it was not possible to establish a relationship between mastication and malnutrition; 6. Reviews, letters, books, congress abstracts, case reports, case series, opinion articles, technical articles, and guidelines; 7. Studies unavailable for full-text reading.

**REFERENCES**

1. Alia S, Aquilanti L, Pugnaloni S, Paolo A Di, Rappelli G, Vignini A. The Influence of Age and Oral Health on Taste Perception in Older Adults: A Case-Control Study. Nutrients [Internet]. 2021;13(11):4166.

2. Aquilanti L, et al. Impact of Elderly Masticatory Performance on Nutritional Status: An Observational Study. Medicina (Kaunas). 2020 Mar;56(3).

3. Baumgartner W, Schimmel M, Müller F. Oral health and dental care of elderly adults dependent on care. Swiss Dent J. 2015;125(4):417–26.

4. Braud A, Lourtioux F, Picouet P, Maitre I. Food-related oral discomfort: A cross-sectional survey assessing the sensory dimension of oral discomfort in French independently living adults. J Oral Rehabil. 2021;48(8):916–26.

5. De Freitas Borges T, Alves Mendes F, Oliveira TRC, Prado CJ, Das Neves FD. Overdenture with immediate load: Mastication and nutrition. Br J Nutr. 2011;105(7):990–4.

6. Brodeur JM, Laurin D, Vallee R, Lachapelle D. Nutrient intake and gastrointestinal disorders related to masticatory performance in the edentulous elderly. J Prosthet Dent. 1993 Nov;70(5):468–73.

7. Chatindiara I, Sheridan N, Kruger M, Wham C. Eating less the logical thing to do? Vulnerability to malnutrition with advancing age: A qualitative study. Appetite [Internet]. 2020;146(June 2019):104502. Available from: https://doi.org/10.1016/j.appet.2019.104502

8. Cousson PY, Bessadet M, Nicolas E, Veyrune JL, Lesourd B, Lassauzay C. Nutritional status, dietary intake and oral quality of life in elderly complete denture wearers. Gerodontology. 2012;29(2):E685–92.

9. Harel J, Fossaert R, Bérard A, Lafargue A, Danet-Lamasou M, Poisson P, et al. Masticatory coefficient and physical functioning in older frail patients admitted for a Comprehensive Gerontological Assessment. Arch Gerontol Geriatr [Internet]. 2021;95.

10. Hutton B, Feine J, Morais J. Is there an association between edentulism and nutritional state? J Can Dent Assoc. 2002 Mar;68(3):182–7.

11. Masanori I, et al. A Two-Year Longitudinal Study of the Association between Oral Frailty and Deteriorating Nutritional Status among Community-Dwelling Older Adults. Int J Environ Res Public Health. 2021;18(1):213.

12. Khandelwal R, Kapil U. Prevalence and factors associated with underweight, overweight and obesity amongst elderly population living at high altitude regions of rural Uttarakhand, India. Ann Nutr Metab. 2017;71(2):1–1433.

13. Khandelwal R, Kapil U, Gupta A. Epidemiological Determinants of Malnutrition Status among Geriatric Population Residing at High Altitude Region of Rural Uttarakhand, India. Proc Nutr Soc. 2020;79(OCE2):2020.

14. Kiesswetter E, Keijser B, Volkert D, Visser M. The association of oral health with body weight within 10 years in community-dwelling older adults. Eur Geriatr Med. 2018;9:1–367.

15. Kim S, Kwon YS, Ki Hwan Hong. What Is the Relationship between the Chewing Ability and Nutritional Status of the Elderly in Korea? Nutrients. 2023 Apr 23;15(9):2042–2.

16. Kim D, Lim H. Association between combinations of nutritional status and quality of life and food purchasing motives among the elderly in South Korea. Health Qual Life Outcomes [Internet]. 2020;18:1–9. Available from: https://www.proquest.com/scholarly-journals/association-between-combinations-nutritional/docview/2414906039/se-2

17. Kiss CM, Besimo C, Ulrich A, Kressig RW. Nutrition and Oral Health in the Elderly . Aktuel Ernahrungsmed [Internet]. 2016;41(1):27–35.

18. Kolb G, Leischker A, Rehmann P, Wöstmann B. Masticatory Function and Nutritional Status . Aktuel Ernahrungsmed [Internet]. 2016;41(4):271–4.

19. Krishna Prasad D, Demeri F, Hegde C, Anupama Prasad D. Nutritional assessment in completely edentulous geriatric patients. World J Dent [Internet]. 2020;11(4):284–6.

20. Kwon SH, Park HR, Lee YM, Kwon SY, Kim OS, Kim HY, et al. Difference in food and nutrient intakes in Korean elderly people according to chewing difficulty: using data from the Korea National Health and Nutrition Examination Survey 2013 (6th). Nutr Res Pract. 2017 Apr;11(2):139–46.

21. Majumder M, Saha I, Chaudhuri D. Assessment of nutritional risk in community-dwelling older adults (65 to 75 years) in Kolkata, India. J Nutr Gerontol Geriatr. 2014;33(2):126–34.

22. Mann T, H: 200-euberger R, Wong H. The association between chewing and swallowing difficulties and nutritional status in older adults. Aust Dent J. 2013 Jun; 58(2): 200-6.

23. Licia M, Iole VOP. Bite Force in Elderly with Full Natural Dentition and Different Rehabilitation Prosthesis. Int J Environ Res Public Health [Internet]. 2021;18(4):1424.

24. Matsuo K. Impact of deterioration of oral health and eating function on the general health and QOL. Aging Med Healthc. 2019;10(0):13.

25. Mbodj EB, Ngom PI, Seck MT, Aïdara AW, Ndiaye C, Dieng L, et al. Relationships between masticatory performance and nutritional state in complete denture wearers. Odontostomatol Trop. 2008 Jun;31(122):20–6.

26. de Morais C, Oliveira B, Afonso C, Lumbers M, Raats M, de Almeida MD V. Nutritional risk of European elderly. Eur J Clin Nutr. 2013 Nov;67(11):1215–9.

27. Musacchio E, Perissinotto E, Binotto P, Sartori L, Silva-Netto F, Zambon S, et al. Tooth loss in the elderly and its association with nutritional status, socio-economic and lifestyle factors. Acta Odontol Scand. 2007 Apr;65(2):78–86.

28. Oliveira TRC. Avaliação nutricional e protética de pacientes senescentes desdentados - estudo comparativo entre pacientes portadores de próteses totais mucoso-suportada-implanto-retidas e próteses totais convencionais. RPG rev. 2001;12(2):255–63.

29. Terezinha R C de Oliveira 1 MLMAF. Association between nutrition and the prosthetic condition in edentulous elderly. Gerodontology. 2005;21(4):205–8.

30. El Osta N, Hennequin M, Tubert-Jeannin S, Abboud Naaman NB, El Osta L, Geahchan N. The pertinence of oral health indicators in nutritional studies in the elderly. Clin Nutr. 2014 Apr;33(2):316–21.

31. El Osta N, El Osta L, Moukaddem F, Papazian T, Saad R, Hennequin M, et al. Impact of implant-supported prostheses on nutritional status and oral health perception in edentulous patients. Clin Nutr ESPEN. 2017;18:49–54.

32. Özsürekci C, Güngör E, Çalişkan H, Ayçiçek G, Halil MG. Chewing function, sarcopenia and malnutrition in elderly patients. Eur Geriatr Med. 2019;10(0):S248.

33. Özsürekci C, Kara M, Güngör AE, Ayçiçek GŞ, Çalışkan H, Doğu BB, et al. Relationship between chewing ability and malnutrition, sarcopenia, and frailty in older adults. Nutrition in Clinical Practice. 2022 Jun 16;37(6):1409–17.

34. Palmer CA. Gerodontic nutrition and dietary counseling for prosthodontic patients. Dent Clin North Am [Internet]. 2003;47(2):355–71.

35. Putri APD, Dewi RS, Maxwell D. Masticatory Ability and Nutritional Status in Elderly Population. J Int Dent Med Res [Internet]. 2020;13(4):1397–404.

36. Riesen M, Chung JP, Pazos E, Budtz-Jørgensen E. Oral care of the elderly. Med Hyg (Geneve). 2022;60(2414):2178–88.

37. Saksono P, Hijryana M, Walls A, Kusdhany L, Indrasari M, Ariani N. Relationships between tooth loss and masticatory performance, nutrition intake, and nutritional status in the elderly. Pesqui Bras Odontopediatria Clin Integr [Internet]. 2019;19(1).

38. Saletti A, Johansson L, Yifter-Lindgren E, Wissing U, Osterberg K, Cederholm T. Nutritional status and a 3-year follow- up in elderly receiving support at home. Gerontology. 2005;51(3):192–8.

39. Samnieng P, Ueno M, Shinada K, Zaitsu T, Wright FAC, Kawaguchi Y. Oral health status and chewing ability is related to mini-nutritional assessment results in an older adult population in Thailand. J Nutr Gerontol Geriatr. 2011;30(3):291– 304.

40. Samnieng P, Ueno M, Shinada K, Zaitsu T, Wright FAC, Kawaguchi Y. Association of hyposalivation with oral function, nutrition and oral health in community-dwelling elderly Thai. Community Dent Health. 2012 Mar;29(1):117–23.

41. Semba RD, Blaum CS, Bartali B, Xue QL, Ricks MO, Guralnik JM, et al. Denture use, malnutrition, frailty, and mortality among older women living in the community. J Nutr Health Aging. 2006;10(2):161–7.

42. Soini H, Routasalo P, Lagstrom H. Nutritional status in cognitively intact older people receiving home care services--a pilot study. J Nutr Health Aging. 2005;9(4):249–53.

43. Svetlana Stajkovic MD, Elizabeth M. Aitken BSc MLIS JH-LM. Unintentional Weight Loss in Older Adults. Am Fam Physician. 2021;104(1):34–40.

44. Tani A, Mizutani S, Kishimoto H, Oku S, Kiyomi I, Chu T, et al. The Impact of Nutrition and Oral Function Exercise on among Community-Dwelling Older People. Nutrients. 2023 Mar 26;15(7):1607–7.

45. Treesattayakul B, Winuprasith T, Theeranuluk B, Trachootham D. Loss of Posterior Occluding Teeth and its Association with Protein-Micronutrients Intake and Muscle Mass Among Thai Elders: A Pilot Study. J Frailty Aging. 2019;8(2):100– 3.

46. Volkert D, Frauenrath C, Kruse W, Oster P, Schlierf G. [Malnutrition in old age--results of the Bethany nutrition study]. Ther Umsch. 1991 May;48(5):312–5.

47. Volkert D, Frauenrath C, Kruse W, Oster P, Schlierf G. Undernutrition in the elderly - what are the causes, what can be done. Ther UMSCHAU. 1991;48(5):312–5.

48. Woo J, Ho S, Lau J, Yuen YK. Chewing difficulties and nutritional status in the elderly. 1994;14(11):1649–54.

49. Yuniendra GG, Rahardjo A, Adiatman M, Maharani DA, Thuy PAV. Relationship between oral health status and masticatory performance with nutritional status in the elderly. J Phys Conf Ser. 2018;1073(4).

50. Wallace M. Factors affecting dietary intake, dietary change, nutritional status and appetite in older adults: impact of oral health status. Queen’s University Belfast; 2020.

51. Ziylan C, Haveman-Nies A, Dongen EJI van, Kremer S, Groot LCPGM de. Dutch nutrition and care professionals experiences with undernutrition awareness, monitoring, and treatment among community-dwelling older adults: a qualitative study. BMC Nutr [Internet]. 2015;1.
